# DRD2 Taq1A Polymorphism-Related Brain Volume Changes in Parkinson's Disease: Voxel-Based Morphometry

**DOI:** 10.1155/2022/8649195

**Published:** 2022-03-28

**Authors:** Kenji Ohira, Hajime Yokota, Shigeki Hirano, Motoi Nishimura, Hiroki Mukai, Takuro Horikoshi, Setsu Sawai, Yoshitaka Yamanaka, Tatsuya Yamamoto, Shingo Kakeda, Satoshi Kuwabara, Tomoaki Tanaka, Takashi Uno

**Affiliations:** ^1^Department of Radiology, Chiba University Hospital, Chiba, Chiba, Japan; ^2^Department of Diagnostic Radiology and Radiation Oncology, Chiba University Graduate School of Medicine, Chiba, Chiba, Japan; ^3^Department of Neurology, Graduate School of Medicine, Chiba University, Chiba, Chiba, Japan; ^4^Division of Laboratory Medicine, Chiba University Hospital, Chiba, Chiba, Japan; ^5^Department of Rehabilitation, Chiba Prefectural University of Health Sciences, Chiba, Chiba, Japan; ^6^Department of Diagnostic Radiology, Hirosaki University Graduate School of Medicine, Hirosaki, Aomori, Japan; ^7^Department of Molecular Diagnosis, Graduate School of Medicine, Chiba University, Chiba, Chiba, Japan

## Abstract

Taq1A polymorphism is a DRD2 gene variant located in an exon of the ANKK1 gene and has an important role in the brain's dopaminergic functions. Some studies have indicated that A1 carriers have an increased risk of developing Parkinson's disease (PD) and show poorer clinical performance than A2 homo carriers. Previous studies have suggested that A1 carriers had fewer dopamine D2 receptors in the caudate and increased cortical activity as a compensatory mechanism. However, there is little information about morphological changes associated with this polymorphism in patients with PD. The study's aim was to investigate the relationship between brain volume and Taq1A polymorphism in PD using voxel-based morphometry (VBM). Based on Taq1A polymorphism, 103 patients with PD were divided into two groups: A1 carriers (A1/A1 and A1/A2) and A2 homo carriers (A2/A2). The volume of the left prefrontal cortex (PFC) was significantly decreased in A2 homo carriers compared to A1 carriers. This finding supports the association between Taq1A polymorphism and brain volume in PD and may explain the compensation of cortical function in A1 carriers with PD.

## 1. Introduction

Parkinson's disease (PD) is a neurodegenerative disease clinically characterized by resting tremor, bradykinesia, muscle rigidity, and posture balance disorder [1]. Although some types of PD, such as familial PD, are caused by a single gene, >90% of cases are sporadic, and it is presumed that both environmental factors and gene polymorphisms can affect the forms of this disease [2].

Dopamine plays an important role in motor control and cognitive functions through interaction with its receptors including dopamine receptor D2 (DRD2). Previous studies showed that DRD2-deficient mice exhibited symptoms that are characteristic of PD [3, 4] and also that DRD2 agonists have been clinically used to improve PD symptoms [5].

The DRD2 gene is located on chromosome 11q22–q23 [6]. The DRD2 Taq1A polymorphism (rs1800497) is one of the DRD2 variants, and the relationship between Taq1A polymorphism and PD has been investigated. Previous studies reported that A1 carriers have an increased risk of developing PD compared to A2 homo carriers [7, 8] and that A1 carriers among patients with PD might be related to clinical symptoms such as motor fluctuations [9], drug-induced hallucinations [10], and impulse control behaviors [11]. The etiology that A1 carriers with PD have poorer clinical performances compared to A2 homo carriers is based on the speculation that the density of DRD2s in the striatum was reduced in A1 carriers compared to A2 homo carriers [12, 13] and that reduced glucose metabolism in many brain regions such as the striatum and prefrontal cortex (PFC) was observed in A1 carriers [14]. However, those results were obtained in healthy subjects (HS) not in patients with PD.

A study using voxel-based morphometry (VBM) showed that the caudate volume was smaller in A1 carriers than in A2 homo carriers in HS [15]. Taq1A polymorphism may also affect the brain morphology in PD, given its effect on dopamine metabolism, but there has been no study regarding the association between Taq1A polymorphism and brain morphology in PD.

The aim of the present study was to determine whether Taq1A polymorphism affects the brain morphology in PD using VBM. In addition, we evaluated the relationship of brain volume differences between HS and patients with PD.

## 2. Materials and Methods

### 2.1. Patients

Our study protocol was approved by the Ethics Review Committee of Chiba University (Approval reference No. 711 and 982). All procedures were carried out in accordance with the relevant guidelines and regulations. Written informed consent was obtained from participants and their parent and/or legal guardian. Overall, 169 patients were recruited at our institute for this study. Patients with a family history of PD, those with PARK2 mutations, and those treated with deep brain stimulation were excluded. Patients who did not undergo three-dimensional T1-weighted imaging on MRI or patients who showed brain tumors, hydrocephalus, traumatic lesions, and cerebrovascular disease on MRI were also excluded.

In total, 103 participants (61 male and 42 female; mean age ± standard deviation (SD): 68.1 ± 10.5 years; mean disease duration 7.4 ± 4.8 years) participated in this study. All patients fulfilled the PD criteria defined by the Movement Disorder Society Clinical Diagnostic Criteria for Parkinson's disease [16]. The patients were evaluated using standard clinical tests, including (i) disease duration, (ii) disease severity according to the Unified Parkinson's Disease Rating Scale (UPDRS) part III [17], (iii) the Mini-Mental State Examination scores (MMSE) [18], and (iv) medication use evaluated as levodopa-equivalent dose (LED) [19]. Patients were evaluated 60–90 min after their usual medications defined at the practical “on” state. 108 HS (48 male and 60 female; mean age ± SD: 65.9 ± 10.1 years) without any mental or neurological disorders were also recruited.

### 2.2. DNA

DNA was extracted from peripheral blood mononuclear cells from blood samples using a MagNA Pure Compact System (Roche Diagnostics, Penzberg, Germany). Genotyping for polymorphisms in DRD2 rs1800497 was performed by directly sequencing regions using High-Resolution Melting (HRM) analysis. Primers for HRM analysis were made using LightCycler Probe Design Software 2.0 (Roche), and the HRM polymerase chain reaction (HRM-PCR) was performed using a LightCycler 480 Instrument (Roche). HRM curve analysis was conducted with LightCycler 480 Gene Scanning Software (version 1.5) [20]. PCR products were purified on a BigDye Xterminator (Life Technologies, Carlsbad, CA) and read by an Applied Biosystems 3130 Genetic Analyzer (Applied Biosystems, Foster City, CA). The A1 carriers were defined as having an AA genotype (15.5%) and AG genotype (59.2%), and the A2 homo carriers were defined as having a GG genotype (25.2%) [21]. The Hardy–Weinberg equilibrium of the rs1800497 distribution had a *p* value of >0.01. We did not perform DNA screening tests on HS.

### 2.3. MRI

All participants underwent MRI scans using a 1.5-T scanner (Siemens Medical System, Erlangen, Germany) or a 3-T scanner (Philips Medical Systems, Best, Netherlands). In this study, three-dimensional T1-weighted imaging was acquired by the following parameters: TR = 7 ms, TE = 3 ms, flip angle = 15°, field of view = 24 cm, section thickness = 1.4 mm, and resolution = 0.9 × 0.9 × 1.0 mm^3^ by the 1.5-T scanner and TR = 8 ms, TE = 3 ms, flip angle = 15°, field of view = 22 cm, section thickness = 1.0 mm, and resolution = 0.9 × 0.9 × 0.7 mm^3^ by the 3-T scanner.

Statistical Parametric Mapping (SPM12, https://www.fil.ion.ucl.ac.uk/spm/software/spm12) was used for image processing for VBM. The 3D-T1 images were segmented into gray matter, white matter, and cerebrospinal fluid images. The Diffeomorphic Anatomical Registration Through Exponential Lie Algebra (DARTEL) toolbox on SPM12 was introduced by Ashburner [22] as an alternative method for precise segmentation and normalization of images. DARTEL normalized these segmented gray and white matter images spatially to the customized template in the standardized anatomic space. The segmented images were modulated by using the Jacobean determinants from the spatial normalization by using DARTEL to preserve the volumes of the gray and white matter in each voxel. An 8 mm Gaussian kernel was used to smoothen the modulated gray matter and white matter images.

### 2.4. Statistics

Analysis of variance (ANOVA) was performed to evaluate differences in age, sex, and total brain volume (TBV) between the A1 carriers, A2 homo carriers, and HS. Chi-square tests were used to compare the proportions of categorical variables, such as sex, between these groups. The Wilcoxon rank sum test was used as a nonparametric test to compare the averages of continuous variables, such as age and TBV, between A1 carriers and A2 homo carriers.

SPM12 software was used to perform the VBM analyses. A full-factorial analysis of covariance (ANCOVA) was used to evaluate morphological differences in gray matter (GM) between groups on a voxel-by-voxel basis throughout the whole brain. Age, gender, MRI scanners, and TBV were added as covariates of no interest to eliminate the effects of confounding factors. The initial voxel threshold was set at 0.001 uncorrected. The cluster-level thresholds were set at *p* < 0.05 corrected for familywise error (FWE). Region of interest (ROI) analyses were also conducted to calculate the caudate, putamen, and globus pallidus (GP) volumes. The each ROI was obtained using FreeSurfer based on a probabilistic Atlas [23, 24]. According to a previous study [25], ROI measurements should be conducted in consideration of the intracranial volume and age. Therefore, the caudate, putamen, and GP volume ratios (each volume divided by TBV) were calculated [26]. The target ROI was also determined as a sphere with a 6-mm radius from the opposite site of the peak coordinate obtained from the results of VBM analyses. ANCOVA was used to compare group differences after adjusting age as covariance. The Bonferroni method was applied for multiple comparisons. We used R version 3.6.3 (R Foundation for Statistical Computing, Vienna, Austria) for statistical analyses except for SPM12. We chose a *p* value of <0.05 to indicate a significant difference.

## 3. Results

### 3.1. Demographic and Clinical Data

We divided the 103 patients into two groups according to their DRD2 Taq1A polymorphism: A1 carriers were 77 (A1/A1: 16, A1/A2: 61) and A2 homo carriers were 26. Fifty-three participants were omitted due to a lack of image data, and 5 participants with organic diseases such as brain infarction were also excluded. Demographic information and clinical data of the participants are shown in Table 1.

There were no significant differences in the distributions of age, sex, and TBV among the A1 carriers, A2 homo carriers, and healthy subjects. The disease duration was longer in A1 carriers than in A2 homo carriers, and the prevalence of dyskinesia tended to be higher in A1 carriers, but the difference was not statistically significant. There were also no significant differences in UPDRS, MMSE, and LED between the A1 carriers and A2 homo carriers (Table 2).

### 3.2. VBM

#### 3.2.1. HS vs. PD

The volume of the left superior temporal gyrus was reduced in patients with PD compared to HS, although no significant difference was detected by multiple comparison correction.

#### 3.2.2. A1 Carriers vs A2 Homo Carriers

**Figure 1:**
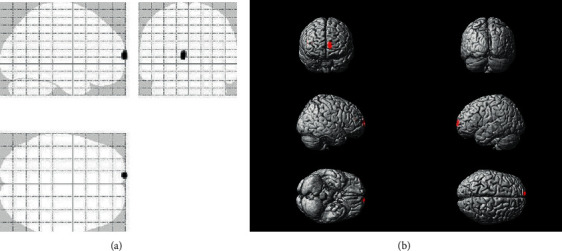
Morphological changes underlying Taq1A polymorphism. The VBM results of 2-dimensional rendering (a) and 3-dimensional (b) overlaid views. The volumes of the left prefrontal lobe were significantly smaller in A2 homo carriers than in A1 carriers.

The volumes of the left PFC were significantly reduced in A2 homo carriers compared to A1 carriers (*t* = 5.00, cluster (*k*) = 102, cluster-level pFWE-corr = 0.009; MNI coordinates of the physical center: −8, 69, and 11) (Figure 1). The volumes of the opposite coordinates from the left PFC were also reduced in A2 homo carriers compared to A1 carriers in the ROI analysis, although no significant difference was detected in multiple comparisons correction. There was also a volume reduction in the left middle frontal gyrus (MFG) in A2 homo carriers, although the voxels did not survive correction for multiple comparisons.

#### 3.2.3. HS vs. A2 Homo Carriers

There were greater volume reductions in the left PFC in A2 homo carriers compared to HS (MNI coordinates of the physical center: −8, 69, and 12), almost the same location as shown in A1 carriers vs A2 homo carriers, although the correction for multiple comparisons showed no significant difference. There was also a volume reduction in the right MFG in A2 homo carriers, although the correction for multiple comparisons showed no significant difference.

#### 3.2.4. HS vs. A1 Carriers

There was no significant difference in volume reduction between HS and A1 carrier patients.

### 3.3. Volume Analysis of Caudate, Putamen, and GP

Caudate, putamen, and GP volume ratios are displayed in Supplementary Table S1. There were no significant differences among A1, A2, and HS in the volume ratios of caudate, putamen, and GP. There was also no significant difference in the volume ratios of these regions with respect to the scanner type (Supplementary Table S2).

## 4. Discussion

Our VBM analysis showed a relationship between brain volume and Taq1A polymorphism in PD patients. The left PFC volume was significantly decreased in A2 homo carriers compared with that in A1 carriers; no significant difference was detected except in the left PFC. ROI analyses of the basal ganglia showed no significant difference between PD patients and HS. The frequency of A1 carriers in the present study seemed relatively high compared with a previous study [15]. However, the A1 allele frequency was 45.1% in our results, which does not seem very different from the Japanese data, in which the frequency was about 42% [27, 28].

There have been no reports regarding the relationship between prefrontal GMV and DRD2 Taq1A polymorphism. However, recent studies have indicated prefrontal atrophic changes in PD which is related to affective and motivational disturbances [27, 28]. A PD group with elevated neuropsychiatric symptoms showed the atrophy in the frontal pole and orbitofrontal cortex in PFC that play an important role in a range of emotion and goal-directed behavior [29]. Another study examined the volume difference between PD and PFC, and it reported that the volume of PFC was decreased in PD with a cognitive decline in MMSE [30]. In our study, there was no significant difference in MMSE between the two groups. However, MMSE may not be a good measure of cognition and cognitive impairment in PD patients, as MMSE could not detect mild cognitive functioning in PD because it does not include tests to assess executive functioning, which is often impaired in the early stages of PD. Montreal Cognitive Assessment (MoCA) [31] has been recommended as the most appropriate screening method for assessing cognitive function in PD [32]. The Frontal Assessment Battery (FAB) may be a useful tool for the screening of executive dysfunction in PD [33]. MoCA or FAB, which was not performed in our study, might have been able to show the relationship between volume differences in PFC and cognitive function that is not detected by MMSE. Although the interaction between prefrontal volume changes and cognitive function or neuropsychiatric symptoms caused by this polymorphism in PD remains an important target for future research; these studies raise the possibility that prefrontal GMV changes may be an indicator for cognitive function or neuropsychiatric symptoms in PD.

The effect of this genetic polymorphism on brain morphology has been reported in various studies [15, 34]. The DRD2 gene might affect brain morphology by modulating the receptor density or function [35]. This speculation was supported by a study showing an association between cerebral morphology and DRD2 density by combined [18F]-fallypride, a high-affinity D2/3 receptor ligand, and voxel-based morphometry [36]. To our knowledge, this is the first study to investigate the association between Taq1A polymorphism and brain morphology in patients with PD. Our study showed an association between the volume of left PFC and DRD2 Taq1A polymorphism in patients with PD. Although the pathogenesis of DRD2 Taq1A polymorphism in PD has not yet been elucidated, considering that the DRD2 gene is expressed in the PFC as well as in the striatum [37–39], it appears to be possible that Taq1A polymorphism may affect the morphological changes in PFC.

A1 carriers of DRD2 Taq1A polymorphism have been associated with reduced DRD2 density in the striatum [12, 13], and varying dopamine levels caused by decreased DRD2 density may affect the brain volume of certain areas by neurotoxic or neurotrophic ways. A previous study using VBM showed that in older HS (i.e., without dementia and PD), A1 carriers had smaller caudate volumes than A2 homo carriers [15]. Another VBM study reported that A1 carriers in HS have reduced midbrain volume, including the substantia nigra [34].

Contrary to our expectations, the left PFC volume was decreased in A2 homo carriers compared to A1 carriers in our study. One possible explanation for this result is that compensatory mechanisms may function in A1 carriers. The motor symptoms of PD do not appear until the dopamine (DA) concentration in the striatum falls below a certain level, approximately 70% [40], and the number of dopaminergic neurons in the substantia nigra is reduced by around 50–60% [41]. The period between the onset of neuronal degeneration and the appearance of symptoms is called the premotor phase, and the absence of motor symptoms during this period is thought to be due to compensatory mechanisms in the brain [42]. VBM studies in PD have also found PD-related brain volume increases in certain brain areas in addition to volume losses in some other regions. The increased brain areas included the frontal lobe, temporoparietal junction, parietal lobe, insula, anterior cingulate cortex, basal ganglia, and thalamus, with these varying depending on the reports [43–46]. It was suggested that PD-related brain volume increase might reflect structural changes that compensate for nigrostriatal dopaminergic pathway dysfunction [47].

Compensatory functions have also been reported in DRD2 Taq1A polymorphism in PD. A functional magnetic resonance imaging (fMRI) study reported that when performing motor tasks, A1 carriers of DRD2 Taq1A polymorphism in PD showed activation in broader brain areas including PFC than A2 homo carriers [48]. The findings that A1 carriers need to activate their motor systems more severely to achieve a complex motor task were interpreted as compensatory mechanisms, for A1 carriers may have a higher risk of developing PD, and A1 carriers with PD are more likely to have poor clinical symptoms [9–11]. Although the precise pathogenesis of DRD2 Taq1A polymorphism in PD remains obscure, the current study demonstrated that DRD2 Taq1A polymorphism was associated with brain volume changes in PD and that cerebral compensatory mechanisms might be present in A1 carriers.

In the VBM analysis, there was a significant volume difference only in the left PFC, not in the right PFC. However, ROI analyses showed a right PFC volume reduction in A2 homo carriers, meaning that volume reduction also occurred in the right PFC, although the number of voxels was not significantly different by multiple comparisons.

Several limitations of this study should be acknowledged. First, we did not analyze DRD2 Taq1A polymorphism in HS. Therefore, we did not determine if the volume difference observed in the left PFC was only due to DRD2 Taq1A polymorphism or if it was caused by the interaction between PD and DRD2 Taq1A polymorphism. Second, as the association between DRD2 Taq1A polymorphism and PD-related clinical symptoms was not determined in our study, the association between the morphological changes caused by Taq1A polymorphism and behavioral performance remains unclear. Third, although there was no significant difference, the disease duration was shorter and the incidence of dyskinesia was lower in A2 homo carriers compared to A1 carriers. We speculated that the reduction in left PFC volume in A2 homo carriers indicated that a compensatory mechanism might work in A1 carriers because of the rather better clinical data in A2 homo carriers. Fourth, we did not analyze the LRRK2 characterized by relatively preserved cognitive functions and a postural instability and gait disorder phenotype [49, 50] and GBA mutations related to higher frequencies of visual hallucinations and a worse cognitive profile [51]. Although we could not exclude the possibility that these variants might have an effect on our result, previous studies have indicated that mutations in the GBA and LRRK2 genes are not important determinants of morphological changes in the brain [52, 53]. Considering these results, we speculate that the LRRK2 and GBA mutations had a small impact on our results. Finally, two models, the 1.5-T MR system and the 3.0-T MR system, were used for our subjects. Although the variability between measurements at 1.5-T and 3-T has different sources, a previous study showed that the volumes would not differ between 1.5-T and 3.0-T [54]. The reason is that SPM12 developed by a unified segmentation method [55] is thought to be robust against inhomogeneity and provides an estimate of the intensity of a tissue class from a fitting of spatial priors to the image, which allows for different image intensities between scans [56]. We also added two MRI models as covariates of no interest to eliminate the effects of confounding factors for the VBM analysis. Harmonization [57] developed for robust pooling and harmonizing imaging data from varying acquisition protocols would be a useful tool for image processing in future studies.

In conclusion, we performed VBM analysis to determine whether Taq1A polymorphism affects the brain morphology in patients with PD. The volumes in the left PFC were decreased in A2 homo carriers compared with those in A1 carriers, perhaps reflecting compensatory mechanisms in A1 carriers. However, the effect of Taq1A polymorphism alone is not able to explain the morphological changes seen in PD patients. Further studies are needed to examine the effects of other gene polymorphisms that may be relevant to the pathogenesis of PD.

## Figures and Tables

**Table 1:** Demographic and clinical characteristics of participants.

	A1 carrier	A2 carrier	HS	*p* value
Number, no	77	26	108	
Female, no (%)	31 (40.3)	11 (42.3)	48 (44.4)	0.851
Age, mean (SD)	67.43 (11.23)	70.00 (7.87)	65.91 (10.09)	0.172
TBV, mean (SD), ml	1049.41 (129.57)	1043.51 (121.92)	1023.19 (98.68)	0.282
3.0 T MRI, no (%)	10 (13)	3 (11.5)	15 (13.9)	0.947

TBV, total brain volume; LED, levodopa-equivalent dose; HS, healthy subjects.

**Table 2:** Demographic and clinical characteristics of PD patients.

	A1 carrier	A2 homo carrier	*p* value
Number, *n* (%)	77 (74.8)	26 (25.2)	
Female, *n* (%)	31 (40.3)	11 (42.3)	1
Age, mean ± SD	67.43 ± 11.23	70.00 ± 7.87	0.283
Disease duration, mean ± SD	7.77 ± 5.40	5.73 ± 3.35	0.074
TBV, mean ± SD, ml	1049.41 ± 129.57	1043.51 ± 121.92	0.839
Dyskinesia, *n* (%)	28 (36.4)	4 (15.4)	0.08
UPDRS, mean ± SD	25.71 ± 16.18	21.96 ± 11.76	0.283
MMSE, mean ± SD	27.73 ± 2.89	27.17 ± 3.13	0.48
Total LED, mean ± SD, mg/day	636.45 ± 372.69	525.57 ± 402.48	0.206

TBV, total brain volume; UPDRS, Unified Parkinson's Disease Rating Scale; MMSE, Mini-Mental State Examination scores; LED, levodopa-equivalent dose.

## Data Availability

The data used to support the findings of the study are included within the article.

## Supplementary Materials

Supplementary Materials Supplementary Table S1: caudate volume ratios. Supplementary Table S2: volume analysis of the putamen and globus pallidus with respect to scanner type (Supplementary Materials).
