# Supplementary material for: DRD2 Taq1A Polymorphism-Related Brain Volume Changes in Parkinson's Disease: Voxel-Based Morphometry
Source: Parkinsons Dis. 2022 Mar 28;2022:8649195. doi: 10.1155/2022/8649195 (PMC8979712; doi:10.1155/2022/8649195)
Supplement: Supplementary Materials — Supplementary Table S1: caudate volume ratios. Supplementary Table S2: volume analysis of the putamen and globus pallidus with respect to scanner type (Supplementary Materials). [file 8649195.f1.docx]

**Supplementary Materials**

**DRD2 Taq1A polymorphism-related brain volume changes in Parkinson’s disease: voxel-based morphometry**

Kenji Ohira, Hajime Yokota, Shigeki Hirano, Motoi Nishimura, Hiroki Mukai, Takuro Horikoshi, Setsu Sawai, Yoshitaka Yamanaka, Tatsuya Yamamoto, Shingo Kakeda, Satoshi Kuwabara, Tomoaki Tanaka and Takashi Uno

**Supplementary Tables:**

**Supplementary Table S1.** Volume analysis of caudate, putamen and GP.

**Supplementary Table S2.** Caudate, putamen, and GP volume ratios with respect to scanner type.

| **Supplementary Table S1. Volume analysis of caudate, putamen and GP.** | | | | |
| --- | --- | --- | --- | --- |
|  | A1 carrier | A2 carrier | HS | p value |
| Caudate | | | | |
| Right (%) | 0.24 (0.04) | 0.24 (0.04) | 0.24 (0.04) | 0.573 |
| Left (%) | 0.23 (0.04) | 0.23 (0.04) | 0.23 (0.03) | 0.288 |
| Putamen | | | | |
| Right (%) | 0.29 (0.04) | 0.28 (0.05) | 0.28 (0.04) | 0.244 |
| Left (%) | 0.28 (0.04) | 0.27 (0.04) | 0.28 (0.04) | 0.312 |
| GP | | | | |
| Right (%) | 0.13 (0.02) | 0.13 (0.02) | 0.13 (0.02) | 0.547 |
| Left (%) | 0.13 (0.02) | 0.13 (0.02) | 0.12 (0.01) | 0.548 |
| Data show mean caudate, putamen and, globus pallidus volume ratios (caudate, putamen and, globus pallidus volume divided by total brain volume) and standard deviations. | | | | |
| GP, globus pallidus; HS, healthy subjects | | | | |

| **Supplementary Table S2. Volume ratios of caudate, putamen, and GP with respect to scanner type.** | | | |
| --- | --- | --- | --- |
|  | 3.0T | 1.5T | p-value |
| 3.0T, no (%) | 28 (13) | 183 (87) |  |
| Caudate | | | |
| Right (%) | 0.24 (0.04) | 0.24 (0.04) | 0.941 |
| Left (%) | 0.23 (0.04) | 0.23 (0.03) | 0.847 |
| Putamen | | | |
| Right (%) | 0.27 (0.04) | 0.27 (0.04) | 0.479 |
| Left (%) | 0.28 (0.04) | 0.28 (0.04) | 0.31 |
| GP | | | |
| Right (%) | 0.13 (0.02) | 0.13 (0.02) | 0.659 |
| Left (%) | 0.12 (0.02) | 0.13 (0.02) | 0.375 |
| The data shows the mean caudate, putamen and GP volume ratios (each volume divided by total brain volume) and standard deviations. | | | |
| GP, globus pallidus | | | |
